# The Relationship Between Sleep Duration and Participation in Home, School, and Community Activities Among School-Aged Children

**DOI:** 10.3389/fnins.2019.00860

**Published:** 2019-08-14

**Authors:** Chi-Wen Chien, Pauline Cheung, Chao-Ying Chen

**Affiliations:** Department of Rehabilitation Sciences, The Hong Kong Polytechnic University, Kowloon, Hong Kong

**Keywords:** participation, sleep, Participation and Environment Measure, PEM-CY, weekday sleep duration, weekend sleep duration

## Abstract

Sleep duration has important implications for children’s participation in daily activities; however, past attempts to examine this relationship has been limited to specific types of physical or educational activities. The present study aimed to investigate the relationship between sleep duration and participation in various daily activities among school-aged children. A school-based sample of 391 children aged 5–12 years (boys: 52.4%) participated in this cross-sectional survey. Sleep duration was quantified using parental reports of their children’s bedtime and wake-up time on weekdays and weekends. The parent-reported Participation and Environment Measure for Children and Youth was used to measure their children’s participation frequency and involvement in 25 home, school, and community activities. The results of hierarchical regression analyses showed that, when the demographic variables were controlled for, weekday sleep duration was positively related to homework involvement and negatively related to the frequency of TV viewing; however, it was unrelated to participation in school and community activities. Conversely, weekend sleep duration was positively related to overall participation in school activities, and participation frequency and involvement in some home and community activities. Furthermore, sleep duration was approximately an hour shorter on weekdays than on weekends. These results suggest that weekend sleep duration has stronger positive implications for children’s participation in daily activities than does weekday sleep duration. Interventions aiming to promote children’s activity participation may either prolong children’s weekend sleep duration or address their shorter weekday sleep duration.

## Introduction

Adequate sleep plays an important role in healthy child development, and it is also required for the maintenance of physical and mental health. According to the National Sleep Foundation (Hirshkowitz et al., 2015), school-aged children (6–13 years) are recommended to sleep for a duration of 9–11 h per night. However, when compared to earlier times, children in modern societies do not get enough sleep; this is especially true for children who live in Asian and North American countries (Matricciani et al., 2012). Insufficient sleep has adverse effects on the cognition, emotional regulation, energy balance, and metabolism of children (St-Onge, 2013; Chaput et al., 2016; Bolinger et al., 2018). These effects have long-term consequences for children’s development (Chaput et al., 2016), academic performance (Curcio et al., 2006), health (Smaldone et al., 2007; Medic et al., 2017), and quality of life (Magee et al., 2017). Therefore, understanding the effects of insufficient sleep on children has important implications for the foundation of health guidelines and the development of intervention strategies that can alleviate the adverse effects of insufficient sleep.

Insufficient sleep among school-aged children may lead to low levels of participation in daily activities or a lack of balanced engagement in a variety of daily activities (Ortega et al., 2011; Engle-Friedman, 2014). However, the existing literature on the relationship between sleep duration and activity participation is primarily limited to specific types of physical or educational activities that have been examined across disparate studies (Li et al., 2014; Jiang et al., 2015; Khan et al., 2015; Lin et al., 2018). Furthermore, these studies did not examine the relationships between sleep duration and the levels of children’s involvement in activities; instead, the participation duration or frequency has been operationalized in most of the studies. The construct of participation has been refined in recent years (Coster et al., 2011b; Kang et al., 2014; Imms et al., 2016b), and it includes two key dimensions: attendance and involvement. Attendance is defined as “being there” reflected by the time spent on or frequency of engaging in activities. Involvement is conceptualized as “the experience of participation” which may include elements of motivation, persistence, social connection, and level of affect (Chien et al., 2014; Imms et al., 2016b). According to this bi-dimensional framework of activity participation, attendance is a necessary but insufficient requirement for high levels of involvement in activities (Imms et al., 2016b). For example, a child may attend soccer practice but may spend a majority of the time passively running around the field and watching for his/her teammates’ play the sport. Therefore, it is important to examine the relationship between sleep duration and both dimensions of activity participation, namely, attendance and involvement.

The objective of the present study was to examine the relationship between sleep duration (during weekdays and the weekend) and two dimensions of activity participation (frequency and involvement) among school-aged children. The activities to be examined in this study pertained to a variety of activities in which children took part at home, at school, and in the community.

## Materials and Methods

### Participants

Participants were recruited from four mainstream primary schools using convenience sampling. First, invitations to participate in this study were sent to all the schools in Hong Kong. Those schools that were first to accept the invitation in each of the four major geographical regions (i.e., Hong Kong Island, Kowloon, New Territory East, and New Territory West) were included in the study. Children who met the following criteria were included in the study sample: (1) between the ages of 5 and 12 years; (2) no history of diagnoses of diseases or disabilities that might interfere with their ability to participate in activities (i.e., as per parent-reports); and (3) parents can read Chinese.

### Procedure

A cross-sectional school-based survey was conducted between 2017 and 2018. An assessment packet that consisted of printed invitation letters, consent forms, and survey questionnaires were distributed to all the children in the four participating schools. Children were instructed to give the packets to their parents. Parents who were willing to participate in the research study were asked to provide written consent, complete the questionnaires, and return them by post using the prepaid envelopes that were included in the assessment packet. Ethical approval for the present study was granted by the ethical review committee of the Hong Kong Polytechnic University.

### Measures

#### Activity Participation

The Participation and Environment Measure for Children and Youth (PEM-CY) (Coster et al., 2011b) is a well-developed parent-reported measure of children’s participation in a range of daily activities. The PEM-CY consists of 25 items that measures children’s participation in a board range of activities at home (10 items), at school (5 items), and in the community (10 items). In each type of activity, there are a few illustrative examples. For example, in the activity of indoor play and games, the examples include playing with toys, puzzles, or board games, playing kitchen, or dress-up. Parents are asked to think about all of the examples that belong to the activity category when answering following questions. First, parents report how often your child has participated in one or more activities of this type over the last 4 months (0 = never; 7 = daily) and, secondly, how involved your child is when participating in one or two activities of this type that he or she does most often (1 = minimally involved; 5 = very involved). Two types of composite participation scores (namely frequency and involvement) can be generated by averaging the individual item scores that have been recorded for each of the three domains of activities (i.e., home, school, and community settings). The PEM-CY has demonstrated fair to high internal consistency (Cronbach’s α = 0.55–0.86) and satisfactory test–retest reliability (intraclass correlation coefficients = 0.70-0.84) (Coster et al., 2011a; Chien et al., 2019).

#### Sleep Duration

Children’s sleep schedules were assessed using a questionnaire that was designed for the purpose of the present study. Parents were asked to indicate the time at which their children typically went to bed at night and woke up in the morning on weekdays and weekends. Sleep duration was calculated as the time that had lapsed between bedtime and wake-up time on weekdays and weekends. The test–retest reliability of this indicator across a 1-month interval was found to be satisfactory (intraclass correlation coefficient = 0.79 for weekdays and 0.81 for weekends) in a subset of 55 children of the study sample.

#### Demographic Characteristics

A demographic questionnaire was used to obtain information about children’s gender, age, and the presence of any diagnoses/ disabilities, as well as the respondent’s relationship with his/her child, age, education, occupation, monthly household income, and district of residence.

### Data Analysis

Means and standard deviations were computed for continuous variables, and percentages were computed for categorical variables. One-way analyses of variance and (independent-samples and paired) *t*-tests were used to examine group differences.

To examine the relationship between sleep duration and activity participation, hierarchical multiple linear regression analyses were conducted. Each of the two composite participation scores for the three domains of activities (i.e., home, school, and community) served as the dependent variable. Since the age, gender, and household income of children have been found to strongly influence activity participation (Brown et al., 2011; Anaby et al., 2014; Imms et al., 2016a), these variables were included as control variables in the first block of the models. In the second block of the models, sleep duration served as the independent variable. Separate analyses were conducted for sleep duration on weekdays and weekends, as different sleep patterns have been reported for these two parts of the week (Wing et al., 2009). The significance level at which the results of these analyses were tested was specified at 0.05.

Furthermore, hierarchical regression analyses were repeated for each PEM-CY item. For these item-level analyses, Bonferroni corrections (Bland and Altman, 1995) were applied to adjust *p*-values to 0.005, 0.010, and 0.005 for participation in home, school, and community activities, respectively.

In addition, variance inflation factors and residual analyses were performed to search for violations of necessary assumptions (e.g., multicollinearity, homescedasticity, normality, and linearity) for the multiple regression analysis (Tabachnik and Fiddel, 2007). We found that the variation inflation factors of all control and independent variables were found to be less than 3, indicating that no multicollinearity was present (Pallant, 2007). The residuals of the children’s participation in each domain of activities or individual activity appeared to be linear, normally distributed, and homoscedastic when the normality probability plots and scatterplots were examined.

## Results

### Demographics, Sleep Duration, and Activity Participation

A total of 517 parents/caregivers (response rate: 34.9%) completed the research questionnaires; of these, 396 (76.6%) were mothers. Further, 82 children had known diagnoses/disabilities and were therefore excluded from the sample. Additionally, 88 children were sibling pairs that represented the same family. Since they tended to coparticipate in activities and have similar sleep schedules, we randomly excluded the data of one child from each family. The final sample included 391 children: 205 boys (52.4%) and 186 girls (47.6%). Their mean age was 8.9 years (*SD* = 1.8), and 272 (69.6%) children’s families had monthly incomes that were equal to or higher than the median.

Sleep duration and activity participation as a function of age, gender, and household income are shown in Table 1. The mean sleep duration on a typical weeknight was 9.02 h, and 58.8% of the children slept for a duration that was lower than the recommended duration for children aged 6–13 years: 9–11 h per night (Hirshkowitz et al., 2015). No significant differences emerged between the groups that differed on gender and household income; however, younger children slept for a longer duration than their older counterparts (*p* < 0.001). In contrast, children slept for a significantly longer duration (*p* < 0.001) on weekend nights (*M* = 10.01 h) than on weeknights. There were 17.4% of children who slept for less than 9 h on weekend nights. Age, gender, and household income did not have a significant effect on weekend sleep duration.

**TABLE 1:** Sleep duration and activity participation as a function of age, gender, and household income.

	**Age (in years)**				**Gender**			**Household monthly income**		
			
	**5.5–7.5**	**7.6–9.5**	**9.6–12.5**	***p***	**Boys**	**Girls**	***p***	**<median**	**≥median**	***p***
	**(*n* = 126)**	**(*n* = 114)**	**(*n* = 151)**		**(*n* = 205)**	**(*n* = 186)**		**(*n* = 118)**	**(*n* = 272)**	
Weekday sleep duration	9.31 (0.68)	8.99 (0.69)	8.81 (0.77)	<0.001	9.06 (7.32)	8.99 (0.76)	0.321	9.03 (0.80)	9.02 (0.72)	0.871
Weekend sleep duration	10.14 (0.87)	10.05 (1.06)	10.02 (1.04)	0.620	10.00 (1.00)	10.14 (0.98)	0.127	10.18 (1.17)	10.02 (0.90)	0.127
**Home participation**										
Frequency	5.46 (0.69)	5.50 (0.64)	5.61 (0.64)	0.139	5.53 (0.64)	5.53 (0.69)	0.986	5.50 (0.74)	5.55 (0.63)	0.487
Involvement	3.89 (0.60)	3.76 (0.61)	3.85 (0.61)	0.274	3.85 (0.62)	3.83 (0.59)	0.754	3.73 (0.64)	3.89 (0.59)	0.021
**School participation**										
Frequency	3.97 (1.17)	4.21 (1.13)	4.83 (0.92)	<0.001	4.31 (1.17)	4.42 (1.09)	0.343	4.24 (1.17)	4.42 (1.11)	0.154
Involvement	3.40 (1.02)	3.63 (0.98)	3.92 (0.90)	<0.001	3.64 (1.03)	3.69 (0.94)	0.630	3.54 (1.05)	3.71 (0.96)	0.120
**Community participation**										
Frequency	2.68 (0.74)	2.45 (0.77)	2.57 (0.88)	0.093	2.60 (0.81)	2.54 (0.80)	0.509	2.33 (0.76)	2.66 (0.80)	<0.001
Involvement	2.89 (0.75)	2.66 (0.92)	2.78 (0.91)	0.146	2.79 (0.86)	2.77 (0.87)	0.823	2.52 (0.97)	2.87 (0.80)	0.001

With regard to activity participation, the average frequency scores were 5.53, 4.36, and 2.57 for the home, school, and community activities, respectively. These values indicate that, on average, children engaged in home, school, and community activities few times a week, once a week, and once a month, respectively. The average involvement scores revealed that parents perceived their children’s involvement in home (*M* = 3.84), school (*M* = 3.67), and community (*M* = 2.78) activities to be of a moderate level. There were no significant age and gender differences in children’s activity participation; however, older children participated more frequently and were more involved in school activities than their younger counterparts (*p* < 0.001; see Table 1). Additionally, children from high-income families participated more frequently in community activities and were more involved in home and community activities than children from low-income families (*p* ≤ 0.021).

### Relationships Between Sleep Duration and Participation in Home Activities

Table 2 shows the relationships that emerged between participation in home activities and sleep duration. With regard to participation frequency, weekday sleep duration was found to be negatively related to watching TV, videos, and DVDs (β = −0.149, *p* = 0.004). On the other hand, with regard to participation involvement, weekday sleep duration was positively related to doing homework (β = 0.184, *p* = 0.001). Furthermore, children who slept for longer duration on weekends tended to be more involved in home activities as a whole (β = 0.119, *p* = 0.022) and specifically in tasks that were involved in preparing school materials at home (β = 0.177, *p* = 0.001).

**TABLE 2:** Relationships, indicated by regression coefficients, between sleep duration and participation in home activities.

	**Weekday sleep duration**			**Weekend sleep duration**		
		
**Dependent variable**	**β**	**95% CI**	***p***	**β**	**95% CI**	***p***
**Participation frequency**						
Composite frequency score	0.025	−0.081, 0.131	0.640	0.069	−0.031, 0.171	0.176
Computer and video games	–0.023	−0.127, 0.082	0.673	–0.053	−0.153, 0.046	0.291
Indoor play and games	0.094	−0.002, 0.191	0.056	–0.004	−0.097, 0.088	0.924
Arts, crafts, music, and hobbies	–0.023	−0.131, 0.085	0.674	0.087	−0.014, 0.189	0.094
Watching TV, videos, and DVDs	**−0.149**	**−0.254, −0.042**	**0.004**	–0.070	−0.171, 0.031	0.176
Getting together with other people	–0.025	−0.132, 0.082	0.641	–0.036	−0.138, 0.064	0.476
Socializing using technology	–0.017	−0.117, 0.082	0.725	0.029	−0.065, 0.124	0.543
Household chores	0.131	0.025, 0.237	0.015	0.104	0.003, 0.206	0.042
Personal care management	–0.014	−0.120, 0.093	0.800	0.121	0.020, 0.222	0.019
School preparation (not homework)	0.046	−0.058, 0.151	0.383	0.078	−0.021, 0.178	0.124
Homework	0.019	−0.087, 0.126	0.721	0.095	−0.006, 0.197	0.065
**Participation involvement**						
Composite involvement score	0.072	−0.036, 0.180	0.191	**0.119**	**0.017, 0.222**	**0.022**
Computer and video games	–0.125	−0.230, −0.019	0.020	–0.064	−0.164, 0.037	0.216
Indoor play and games	0.013	−0.089, 0.117	0.796	0.017	−0.081, 0.115	0.733
Arts, crafts, music, and hobbies	0.037	−0.071, 0.144	0.504	0.065	−0.036, 0.168	0.210
Watching TV, videos, and DVDs	–0.105	−0.003, 0.388	0.053	0.014	−0.087, 0.116	0.782
Getting together with other people	0.089	−0.017, 0.196	0.100	0.061	−0.040, 0.163	0.236
Socializing using technology	–0.005	−0.109, 0.099	0.921	0.038	−0.060, 0.137	0.449
Household chores	0.053	−0.055, 0.161	0.336	0.100	−0.001, 0.203	0.053
Personal care management	0.055	−0.053, 0.163	0.320	0.082	−0.021, 0.184	0.119
School preparation (not homework)	0.122	0.014, 0.229	0.026	**0.177**	**0.007, 0.279**	**0.001**
Homework	**0.184**	**0.077, 0.291**	**0.001**	0.119	−0.017, 0.222	0.022

*All models were adjusted for children’s age, gender, and household income. Statistically significant results are presented in boldface; *p* < 0.050 and *p* < 0.005 were the levels of significance that were used to test the composite scores and individual item scores, respectively.*

### Relationships Between Sleep Duration and Participation in School Activities

Table 3 shows the relationships that emerged between participation in school activities and sleep duration. Weekday sleep duration was unrelated to participation frequency and involvement in school activities. In contrast, weekend sleep duration was positively related to both the frequency of participation (β = 0.109, *p* = 0.024) and involvement (β = 0.115, *p* = 0.023) in school activities (i.e., composite scale score). Specifically, children who slept for longer duration on weekends tended to participate more frequently (β = 0.154, *p* = 0.002) and be more involved (β = 0.152, *p* = 0.002) in prosocial activities at school by playing roles such as a class leader, discipline leader, or student mentor.

**TABLE 3:** Relationships, indicated by regression coefficients, between sleep duration and participation in school activities.

	**Weekday sleep duration**			**Weekend sleep duration**		
		
**Dependent variable**	**β**	**95% CI**	***p***	**β**	**95% CI**	***p***
**Participation frequency**						
Composite frequency score	0.036	−0.064, 0.136	0.479	**0.109**	**0.014, 0.203**	**0.024**
Classroom activities	0.073	−0.033, 0.180	0.176	0.026	−0.076, 0.128	0.613
Field trips and school events	0.122	0.016, 0.228	0.023	0.046	−0.056, 0.147	0.375
School-sponsored teams, clubs and organizations	–0.053	−0.156, 0.049	0.308	0.018	−0.079, 0.116	0.714
Getting together with peers outside of class	0.073	−0.034, 0.180	0.184	0.052	−0.050, 0.155	0.316
Special roles at school	<0.001	−0.102, 0.103	0.999	**0.154**	**0.057, 0.251**	**0.002**
**Participation involvement**						
Composite involvement score	–0.013	−0.118, 0.091	0.804	**0.115**	**0.015, 0.214**	**0.023**
Classroom activities	0.054	−0.053, 0.163	0.321	0.121	0.017, 0.223	0.021
Field trips and school events	–0.049	−0.157, 0.059	0.370	0.044	−0.058, 0.147	0.393
School-sponsored teams, clubs and organizations	–0.021	−0.127, 0.084	0.690	0.039	−0.061, 0.139	0.447
Getting together with peers outside of class	0.017	−0.092, 0.126	0.765	0.006	−0.098, 0.110	0.910
Special roles at school	0.021	−0.086, 0.127	0.704	**0.152**	**0.052, 0.252**	**0.002**

*All models were adjusted for children’s age, gender, and household income. Statistically significant results are presented in boldface; *p* < 0.050 and *p* < 0.010 were the levels of significance that were used to test the composite scores and individual item scores, respectively.*

### Relationships Between Sleep Duration and Participation in Community Activities

Table 4 shows the relationships that emerged between participation in community activities and sleep duration. Weekday sleep duration was unrelated to both participation frequency and involvement in community activities. Similarly, weekend sleep duration was also unrelated to the level of involvement in community activities; however, it was positively related to the frequency of participation in community-based classes and lessons (β = 0.149, *p* = 0.003).

**TABLE 4:** Relationships, indicated by regression coefficients, between sleep duration and participation in community activities.

	**Weekday sleep duration**			**Weekend sleep duration**		
		
**Dependent variable**	**β**	**95% CI**	***p***	**β**	**95% CI**	***p***
**Participation frequency**						
Composite frequency score	0.084	−0.024, 0.207	0.122	0.099	−0.002, 0.201	0.057
Neighborhood outings	0.021	−0.008, 0.124	0.690	0.051	−0.046, 0.150	0.299
Community events	–0.041	−0.150, 0.067	0.451	0.090	−0.012, 0.193	0.083
Organized physical activities	0.075	−0.031, 0.181	0.165	0.088	−0.012, 0.189	0.084
Unstructured physical activities	0.069	−0.038, 0.175	0.208	–0.002	−0.104, 0.100	0.977
Classes and lessons (not school-sponsored)	–0.001	−0.106, 0.103	0.983	**0.149**	**0.050, 0.247**	**0.003**
Organizations, groups, clubs, and volunteer or leadership activities	0.003	−0.103, 0.111	0.947	–0.003	−0.105, 0.098	0.950
Religious or spiritual gatherings and activities	0.088	−0.020, 0.196	0.109	–0.015	−0.118, 0.088	0.775
Getting together with other children in the community	0.082	−0.024, 0.187	0.128	0.029	−0.071, 0.129	0.571
Working for pay	0.101	−0.007, 0.210	0.069	–0.021	−0.125, 0.083	0.687
Overnight visits or trips	0.102	−0.006, 0.210	0.066	0.048	−0.056, 0.151	0.362
**Participation involvement**						
Composite involvement score	0.044	−0.067, 0.155	0.431	0.043	−0.063, 0.148	0.428
Neighborhood outings	–0.033	−0.141, 0.075	0.549	0.005	−0.097, 0.108	0.919
Community events	–0.067	−0.177, 0.043	0.234	0.052	−0.052, 0.157	0.327
Organized physical activities	0.120	0.011, 0.228	0.031	0.089	−0.014, 0.192	0.090
Unstructured physical activities	0.058	−0.049, 0.167	0.288	–0.018	−0.121, 0.084	0.726
Classes and lessons (not school-sponsored)	0.038	−0.069, 0.145	0.482	0.077	−0.024, 0.179	0.135
Organizations, groups, clubs, and volunteer or leadership activities	–0.009	−0.119, 0.100	0.863	–0.004	−0.108, 0.100	0.935
Religious or spiritual gatherings and activities	0.009	−0.019, 0.203	0.103	–0.053	−0.159, 0.053	0.323
Getting together with other children in the community	–0.016	−0.124, 0.092	0.775	0.016	−0.087, 0.119	0.759
Working for pay	0.079	−0.030, 0.189	0.157	–0.004	−0.109, 0.101	0.936
Overnight visits or trips	0.045	−0.065, 0.157	0.421	0.064	−0.040, 0.170	0.228

*All models were adjusted for children’s age, gender, and household income. Statistically significant results are presented in boldface; *p* < 0.050 and *p* < 0.005 were the levels of significance that were used to test the composite scores and individual item scores, respectively.*

## Discussion

Activity participation offers children opportunities to learn new skills, develop physical abilities, make friends, and establish their sense of purpose (Law, 2002; Hoogsteen and Woodgate, 2010; Chien and Rodger, 2011), and it has been recognized as an important contributor to health and quality of life (Colver, 2009; Holder et al., 2009; Berg et al., 2018). To our knowledge, the present study is the first to investigate the relationships between sleep duration and the frequency and involvement with which school-aged children participate in a wide range of activities. Overall, our results show that children slept for longer duration of time on weekend nights than on weeknights. Furthermore, longer weekend sleep duration was related to a higher frequency and greater involvement in home, school, and community activities. However, weekday sleep duration was negatively related to the frequency of watching TV and positively related to the involvement in doing homework; it was unrelated to school and community activities. These findings highlight the influence of weekday and weekend sleep duration on children’s different activity participation patterns.

Previous studies have reported that children sleeping less were likely to have a sedentary and inactive lifestyle coupled with more time spent on watching TV and homework completion (Li et al., 2014; Jiang et al., 2015; Lin et al., 2018). Reciprocally, more time spent on TV viewing and finishing homework may delay children’s bedtime and shorten their sleep duration (Li et al., 2007; Calamaro et al., 2009). Similar relationships between weekday sleep duration and the frequency of TV viewing was also observed in the present study, although the cross-sectional research design of this study did not allow us to determinate the direction of the relationships. However, with regard to homework, we found that short sleep duration was associated with low involvement but not high frequency. Thus, we speculated that short sleep duration might lead to tiredness or poor concentration during the daytime (O’Brien, 2009; St-Onge, 2013; Bolinger et al., 2018). This in turn might cause children to be less involved in homework activities and subsequently prolong the time that is required to finish their homework. Because previous studies have focused exclusively on the time that was spent on homework (Li et al., 2014; Jiang et al., 2015), the present findings on the relationship between sleep duration and involvement in doing one’s homework make a noteworthy contribution to the existing literature.

Interestingly, we did not find a relationship between weekday sleep duration and participation in school or community activities. However, it is noted that we controlled for age, gender, and household income when examining the relationships between sleep duration and activity participation. Significant age differences emerged for school participation; similarly, significant differences in participation in community activities emerged between the groups that differed in household income (see Table 1). It is thus possible that the impact of short sleep duration on participation in school and community activities might be largely explained by the demographic variables. To test this possibility, we conducted the additional regression analyses by entering weekday sleep duration first and demographic variables subsequently. Significant relationships between weekday sleep duration and participation in some school and community activities attenuated when the demographic variables were entered into the models (results are not shown). In particular, children’s age and/or household income became the strongest factors of nearly half the number of school and community activities in those models. This result is in accordance with past findings that children’s activity participation involves a dynamic interdependence between personal, familial, and environmental factors (Law et al., 2007; Verdonschot et al., 2009; Anaby et al., 2014; Chien et al., 2017; Cho et al., 2018). Accordingly, weekday sleep duration may play a less important role than other personal and environmental factors in children’s participation in school and community activities.

In contradistinction to the findings that emerged for weekday sleep duration, weekend sleep duration was positively related to participation in not only home activities but also in school and community activities. One possible explanation for the relationships that emerged for weekend sleep duration but not for weekday sleep duration may pertain to the benefits of weekend sleep extension (also known as weekend catch-up sleep). In the present study, the average duration of sleep was approximately an hour (1.04 h) longer on weekends than on weekdays. Particularly, 94.3% of 230 children who slept for shorter duration (i.e., ≤9 h) during the week were found to sleep, on average, 1.38 h (*SD* = 0.97) longer on weekends. Similarly, the longer duration of sleep on weekends than on weekdays (*M* = 0.55; *SD* = 1.08) was also found in 67.7% of 161 children who slept for more than 9 h during the week. This trend is consistent with past findings that elementary-school children (*N* = 5159) slept for longer duration on weekends to compensate for the insufficient sleep that they got throughout the week (Wing et al., 2009). Studies have shown that weekend catch-up sleep can restore the cognitive impairments that were caused by sleep restrictions that one got on weeknights (Kuula et al., 2015; Agostini et al., 2017). Children who slept for longer duration on weekends might have better attention and concentration and, therefore, might demonstrate a higher frequency of participation or greater involvement in activities such as preparing school materials at home, playing special roles at school, and attending extracurricular classes in the community. In the short term, weekend catch-up sleep may serve as a buffer against insufficient sleep on weekdays and may enhance participation in daily activities. Nonetheless, oversleeping on weekends may not be a suitable long-term solution. Specifically, a growing body of research suggests that children who sleep for longer duration on weekends tend to skip breakfast, develop irregular cortisol circadian rhythm, and have impaired executive functions; these in turn result in poor academic performance, depression, and weight gain (overweight/obesity) (Kuula et al., 2015; Becker et al., 2017; Sun et al., 2019).

There are several limitations that must be considered when the findings of the present study are interpreted. First, the present study used a well-developed and validated parent-reported questionnaire (i.e., the PEM-CY) to assess children’s activity participation. However, parents’ interpretations of their child’s participation may differ from those of the children. Furthermore, the PEM-CY did not obtain parents’ report on their children’s participation duration for each activity, and it is possible that children’s sleep duration might be affected more by the activities in which they do not participate often but spend many hours each time they participate. Second, children’s sleep duration was defined as the span of time between bedtime and wake-up time, both of which were reported by parents; therefore, the sleep duration that was computed may be overestimated. In addition, we did not collect either information on daytime naps or sleep quality in this study. Future studies that use objective actigraphic measures of sleep duration and comprehensive measures of sleep parameters are needed. Third, factors such as commuting time, seasonal effects, and environmental barriers that may impact children’s activity participation and sleep behaviors were not taken into account in the present study. Future studies to include these factors may provide us a more comprehensive picture to address the concerns proposed in the present study. Finally, the present study entailed a cross-sectional survey design and, therefore, the causality of the observed relationships must be tested in future prospective and experimental studies.

## Conclusion

Weekday sleep duration was associated with school-aged children’s participation in some home activities but not school or community activities. In contrast, weekend sleep duration was associated with participation frequency and involvement in home, school, and community activities. Our findings also showed that the duration of sleep was approximately an hour longer on weekends than on weekdays, thereby indicating the phenomenon of weekend catch-up sleep that is common among school-aged children. Therefore, interventions that aim to promote children’s participation in daily activities may need to incorporate strategies that ensure the adequate sleep children get on both weeknights and weekend nights. Increasing the awareness of the associations between sleep duration and activity participation could be also the focus of future health promotion programs for school-aged children and their parents.

## Data Availability

The raw data supporting the conclusions of this manuscript will be made available by the authors, without undue reservation, to any qualified researcher.

## Author Contributions

C-WC conceived and designed the study, supervised the data collection, carried out the initial analyses, and drafted the initial manuscript. PC reviewed the relevant literature, carried out parts of the analyses, and critically reviewed the manuscript for important intellectual content. C-YC co-designed the study, reviewed the relevant literature, and critically reviewed the manuscript for important intellectual content. All authors approved the final manuscript as submitted and agreed to be accountable for all aspects of the work.

## Conflict of Interest Statement

The authors declare that the research was conducted in the absence of any commercial or financial relationships that could be construed as a potential conflict of interest.
